# Catheterization of Pulmonary and Carotid Arteries for Concurrent Measurement of Mean Pulmonary and Systemic Arterial Pressure in Rat Models of Pulmonary Arterial Hypertension

**DOI:** 10.21769/BioProtoc.4737

**Published:** 2023-08-20

**Authors:** Tanoy Sarkar, Ayman Isbatan, Sakib M. Moinuddin, Jiwang Chen, Fakhrul Ahsan

**Affiliations:** 1 Department of Pharmaceutical and Biomedical Sciences, California Northstate University College of Pharmacy, Elk Grove, USA; 2 Cardiovascular Research Center, University of Illinois at Chicago, Chicago, IL, USA; 3 Department of Medicine, Section of Pulmonary, Critical Care Medicine, Sleep and Allergy, University of Illinois at Chicago, Chicago, IL, USA

**Keywords:** Pulmonary hypertension, Right heart catheterization, Rat model, Mean pulmonary arterial pressure, Mean systemic arterial pressure, PowerLab system, Pulmonary artery catheterization, Pulmonary artery catheter, Pulmonary artery wedge pressure, Invasive monitoring, Hemodynamic monitoring

## Abstract

Pulmonary hypertension (PH) is a group of pulmonary vascular disorders in which mean pulmonary arterial pressure (mPAP) becomes abnormally high because of various pathological conditions, including remodeling of the pulmonary arteries, lung and heart disorders, or congenital conditions. Various animal models, including mouse and rat models, have been used to recapitulate elevated mPAP observed in PH patients. However, the measurement and recording of mPAP and mean systemic arterial pressure (mSAP) in small animals require microsurgical procedures and a sophisticated data acquisition system. In this paper, we describe the surgical procedures for right heart catheterizations (RHC) to measure mPAP in rats. We also explain the catheterization of the carotid artery for simultaneous measurement of mPAP and mSAP using the PowerLab Data Acquisition system. We enumerate the surgical steps involved in exposing the jugular vein and the carotid artery for catheterizing these two blood vessels. We list the tools used for microsurgery in rats, describe the methods for preparing catheters, and illustrate the process for inserting the catheters in the pulmonary and carotid arteries. Finally, we delineate the steps involved in the calibration and setup of the PowerLab system for recording both mPAP and mSAP. This is the first protocol wherein we meticulously explain the surgical procedures for RHC in rats and the recording of mPAP and mSAP. We believe this protocol will be essential for PH research. Investigators with little training in animal handling can reproduce this microsurgical procedure for RHC in rats and measure mPAP and mSAP in rat models of PH. Further, this protocol is likely to help master RHC in rats that are performed for other conditions, such as heart failure, congenital heart disease, heart valve disorders, and heart transplantation.

## Background

Pulmonary hypertension (PH) encompasses a group of pulmonary vascular disorders with varying etiologies. Depending on the cause and site of the pathogenesis, PH is classified into five groups and many subgroups (Sahay, 2019; Sarah et al., 2021). Despite the differences in the pathological basis of various groups of PH, the disease shares a common definition: a pathological condition in which the mean pulmonary arterial pressure (mPAP) is greater than 25 mmHg at rest or 30 mmHg during exercise (Galiè et al., 2016; Simonneau et al., 2019). Of the five groups of PH, pulmonary arterial hypertension (PAH) and its various subgroups fall in Group I (Wijeratne et al., 2018). In the case of PAH, pulmonary arteries and arterioles undergo pathological changes and thus become thicker and stiffer, resulting in elevated mPAP (Lai et al., 2014; Lan et al., 2018). In other forms of PH (Groups II–V), lung or heart diseases or other conditions increase mPAP (Galiè et al., 2016; Simonneau and Hoeper, 2019).

**Figure 1.**
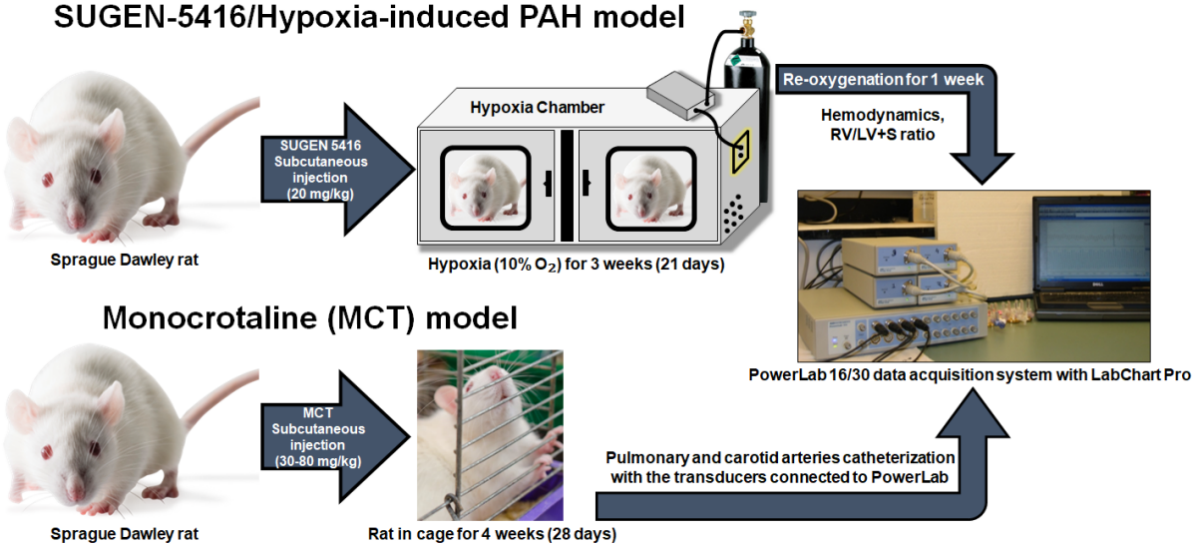
Sugen-5416/Hypoxia-induced pulmonary arterial hypertension (PAH) model and monocrotaline (MCT) model. For the development of Sugen/hypoxia-based models, animals are first treated with a subcutaneous injection of 20 mg/kg Sugen 5416 and then are housed in 10% oxygen for 3–4 weeks. A right heart catheterization (RHC) is performed to measure mean pulmonary arterial pressure (mPAP) soon after the removal of the animals from the hypoxic environment or after three weeks of hypoxia followed by one week of normoxia. In case of monocrotaline-based models, animals receive a single subcutaneous injection of 30–80 mg/kg monocrotaline. The mPAP is measured 3–4 weeks after MCT injection.

Because the chief manifestation of PH is increased mPAP, animal models for PH are developed to recapitulate elevated mPAP (Liu and Yan, 2022; Wu et al., 2022). Of the various animal models for PH, Sugen/hypoxia and monocrotaline-induced rat models of PH are two commonly used models (Stenmark et al., 2009; Vitali et al., 2014; Sztuka and Jasinska-Stroschein, 2017) (Figure 1). To develop hypoxia-based models, animals are first treated with a subcutaneous injection of 20 mg/kg Sugen 5416, a vascular endothelial growth factor receptor antagonist. Then, they are housed in 10% oxygen for 3–4 weeks (Bhat et al., 2017; Honda et al., 2020). A right heart catheterization (RHC) is performed to measure mPAP soon after the removal of the animals from the hypoxic environment or after three weeks of hypoxia followed by one week of normoxia (V. Gupta et al., 2013; Nahar et al., 2014; N. Gupta et al., 2015 and 2017; Nahar et al., 2016; Rashid et al., 2017, 2018a, 2018b and 2019; Keshavarz et al., 2019; Al-Hilal et al., 2021). In the case of monocrotaline-based models, animals receive a single subcutaneous injection of 30–80 mg/kg monocrotaline (Lachant et al., 2018; Jing et al., 2020), an alkaloid that causes PH by injuring pulmonary arterial endothelium (Xiao et al., 2017). The mPAP is measured 3–4 weeks after injection (Gomez-Arroyo et al., 2012; N. Gupta et al., 2017). In both models, mPAP rises to as high as 40–60 mmHg.

In fact, over the past several decades, these models have been used for studying PH pathophysiology and investigating the efficacy of drugs in reducing mPAP (V. Gupta et al., 2013; Maarman et al., 2013; Nahar et al., 2014; N. Gupta et al., 2015 and 2017; Nahar et al., 2016; Rashid et al., 2017, 2018a, 2018b and 2019; Keshavarz et al., 2019; Al-Hilal et al., 2021; Dignam et al., 2022). For mechanistic studies or to evaluate drugs’ efficacy in ameliorating pathological alterations, pulmonary arteries/arterioles are collected, and various histopathological studies are performed. Further, PH is treated with vasodilators to reduce elevated mPAP (Rose-Jones and McLaughlin, 2015). Reduced mPAP increases survival by reducing right ventricular hypertrophy, the chief cause of death in PH patients (Ryan et al., 2015; Zhu et al., 2019; Benza et al., 2022). However, vasodilator therapy also reduces blood pressure, resulting in peripheral hypotension–induced side effects (Packer, 1985; Rich, 2009; Humbert et al., 2014; Chida-Nagai et al., 2020). Thus, for studying the efficacy of investigational and commercially available drugs in animal models of PH, mPAP is measured as a functional endpoint for therapeutic outcomes (V. Gupta et al., 2013; Maarman et al., 2013; Nahar et al., 2014; N. Gupta et al., 2015 and 2017; Nahar et al., 2016; Rashid et al., 2017, 2018a, 2018b and 2019; Keshavarz et al., 2019; Al-Hilal et al., 2021; Beckmann et al., 2022; Dignam et al., 2022). Mean system arterial pressure (mSAP) is also measured to evaluate the influence of the drug on peripheral blood pressure or to assess whether the drug or formulation has pulmonary selectivity, by comparing the extent of reduction in mPAP and mSAP in the same rats. In a series of studies, we have shown that pulmonary selectivity can be evaluated by comparing mPAP and mSAP upon administration of various drug therapies to PH rats (V. Gupta et al., 2013; Maarman et al., 2013; Nahar et al., 2014; N. Gupta et al., 2015 and 2017; Nahar et al., 2016; Rashid et al., 2017, 2018a, 2018b and 2019; Keshavarz et al., 2019; Al-Hilal et al., 2021; Dignam et al., 2022).

To measure mPAP, an RHC is performed to place the catheter in the pulmonary artery. Likewise, for mSAP, a catheter is placed in the carotid artery of the same rats that underwent RHC. In our published studies, we inserted polyethylene (PE) catheters in both pulmonary and carotid arteries (N. Gupta et al., 2014; Nahar et al., 2014; Rashid et al., 2018b). However, insertion of PE catheter in pulmonary arteries is challenging because catheters require a specialized curvature for maneuvering from the jugular vein through the right atrium and right ventricle and finally to the pulmonary artery. Further, exposure to the jugular vein for RHC and carotid artery requires microsurgical procedures and specialized tools. Similarly, recording mPAP and mSAP entails using a specialized instrument. PowerLab, a system connected to bridge amplifiers and equipped with Lab Chart Pro software, is universally used to record mPAP and mSAP (Doggett et al., 2018). However, this system must be calibrated and set up accurately.

While we and others have been performing RHC to measure mPAP and mSAP using PowerLab systems for many years now (Rey et al., 2012; V. Gupta et al., 2013; Nahar et al., 2014; N. Gupta et al., 2015 and 2017; Nahar et al., 2016; Rashid et al., 2017, 2018a, 2018b and 2019; Keshavarz et al., 2019; Al-Hilal et al., 2021), there are no published protocols that list the surgical tools, explain the steps for preparation and insertion of catheters, and describe the setup and calibration of PowerLab lab data acquisition systems. In the absence of such a protocol, a new investigator may take months to establish the methodologies for RHC in rats and recording of mPAP and mSAP using the PowerLab system. Importantly, the unavailability of a thorough protocol is a major reason for significant delays in training new lab personnel in RHC or the transfer of methods from one investigator to another in the same lab. Thus, there is an important need for a detailed protocol for performing RHC in rats and measuring such; in this protocol paper, we put together all steps and processes involved in RHC and measurement of mPAP and mSAP, so that investigators with no training can perform the surgery and record mPAP and mSAP. This protocol can also be deployed in performing RHC in animal models for heart failure, congenital heart disease, heart valve disorders, and heart transplantation.

## Materials and reagents

1. Air-Tite^TM^ All-Plastic Henke-Ject^TM^ syringes (Fisher Scientific, catalog number: 14-817-25)
2. Stainless-steel flat instrument tray (Medicus Health, catalog number: 2841M1)
3. Mayo scissors, straight (Roboz, catalog number: RS-6872) (Figure 2A)
4. Surgical ophthalmic Westcott tenotomy scissors (Codman, catalog number: 28 54-6513) (Figure 2B)
5. Vannas spring scissors (Fine Science Tools, catalog number: 91500-09) (Figure 2C)
6. Vannas micro dissecting spring scissors (Roboz, catalog number: RS-5640) (Figure 2D)
7. Angled vessel cannulation forceps (Fine Science Tools, catalog number: 18403-11) (Figure 2E)
8. Serrated Semken forceps (Fine Science Tools, catalog number: 11008-13) (Figure 2F)
9. Extra fine Graefe forceps, quantity: 2 (Fine Science Tools, catalog number: 11150-10) (Figure 2G)
10. Graefe forceps, quantity: 2 (Roboz, catalog number: RS-5138) (Figure 2H)
11. Delicate suture tying forceps (Fine Science Tools, catalog number: 11063-07) (Figure 2I)
12. Micro serrefines (Fine Science Tools, catalog number: 18055-01) (Figure 2J)
13. Vein pick (Braintree Scientific Inc., catalog number: V-PIC) (Figure 2K)
14. 25 G needle (Sigma Aldrich, catalog number: Z192406) (Figure 2L)
15. 16 G needle (Becton Dickinson, catalog number: 305198)
16. HSW syringe 1 mL capacity, polypropylene (Grainger, catalog number: 45UC66)
17. Silk sutures, non-absorbable, 5-0 (Braintree Scientific, catalog number: SUT-S-106)
18. PE-50 catheter: polyethylene .023" × .038" per ft. (Braintree Scientific, catalog number: PE50)
19. Umbili-Cath^TM^ 3.5 French single lumen polyurethane umbilical vessel catheter (UVC) (Utah Medical Products, catalog number: 4183505)
20. Surgical stainless-steel suture (Ethicon, catalog number: DS24)
21. Acrylic sheet, 6 in. × 6 in., 0.25 inch thick (McMaster-Carr, catalog number: 8560K358)
22. Umbili-Cath^TM^ 3.5 French dual lumen silicone UVC, marked to 34 cm, 20/23 gauge (Utah Medical Products, catalog number: 4273505)
23. Safelet IV catheter 20 gauge 1" Luer tapered end Teflon (Henry Schein Medical, catalog number: 1198184)
24. High temperature cautery kit (Fine Science Tools, catalog number: 18010-00)
25. 3M^TM^ Micropore^TM^ surgical paper tape (Fisher Scientific, catalog number: 19-027761)
26. Betadine^®^ microbicide solution (Fisher Scientific, catalog number: 19-027136)
27. Gauze pad (Fisher Scientific, catalog number: 22-362178)
28. Sprague Dawley rats (Charles River Laboratory, stock number: 400SASSD)
29. Deionized water (Barnstead Mega-Pure D2, Thermo Scientific)
30. Sodium chloride, 0.9% (w/v), isotonic saline, Ricca Chemical (Fisher Scientific, catalog number: 7647-14-5)
31. 99% isopropyl alcohol, IPA (VWR, catalog number: IX0235)
32. Isoflurane liquid (Pharmacompass, NDC: 66794-017-10)
33. Ketamine hydrochloride, 100 mg/mL (Covetrus, NDC: 11695-0703-1)
34. Xylazine 20 mg/mL (Heartland Veterinary Supply and Pharmacy, catalog number: 343720-RX, NDC: 59399-110-20)
  The cannulation tools shown in Figure 2 are listed in detail in the Materials and reagents section.
  **Figure 2.**
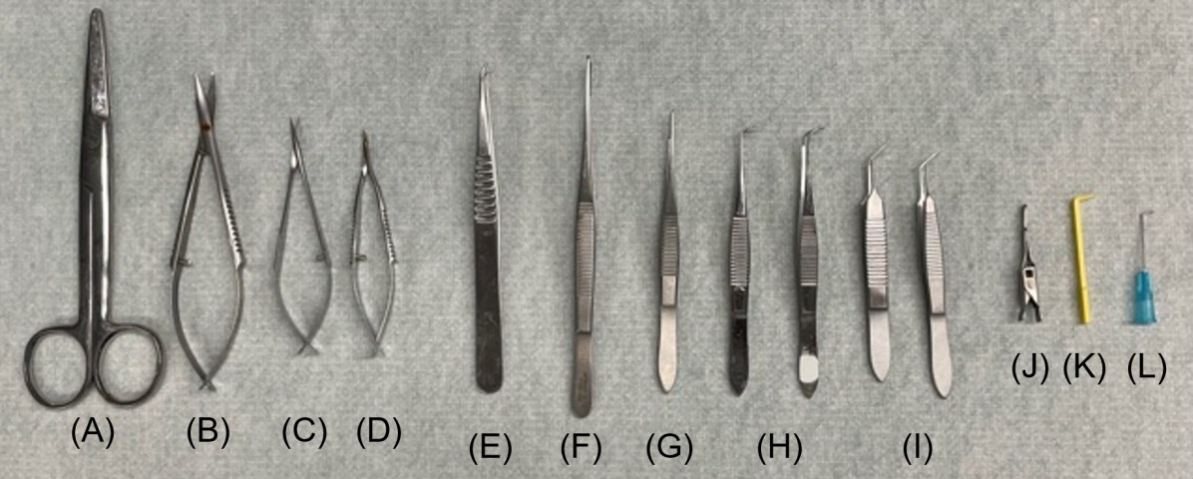
Canulation tools. (A) Mayo scissors; straight, (B) surgical ophthalmic Westcott tenotomy scissors, (C) Vannas spring scissors, (D) Vannas micro dissecting spring scissors, (E) angled vessel cannulation forceps, (F) serrated Semken forceps, (G) extra fine Graefe forceps, (H) Graefe forceps, quantity: 2, (I) delicate suture tying forceps, quantity: 2, (J) micro serrefines, (K) vein pick, (L) 25 G needle.

## Equipment

1. Windows desktop computer
2. PowerLab ML880 16 Channel (ADInstruments)
3. Bridge Amplifier ML221 (ADInstruments)
4. Anesthesia induction chamber (Harvard Apparatus, catalog number:75-2030)
5. Wahl^®^ Trimmer Combo kit (Kent Scientific, catalog number: CL9990-KIT)
6. SP844 medical pressure transducer sensor (Memscap)
7. Blood pressure transducer cable kit MLT1199 (Harvard Apparatus, catalog number: 77-0124)
8. Disposable clip-on BP domes (AD Instruments, catalog number: MLA844)
9. Blue 3-way stopcock, 2 female Luer locks, swivel male Luer lock (Qosina, catalog number: 99740)
10. 3.5×–90× trinocular stereo zoom inverted light microscope (Amscope, catalog number: SM-3TZ-54S-5M)
11. Red 3-way stopcock, 2 female Luer locks, swivel male Luer lock (Qosina, catalog number: 99761)
12. Delta-Cal pressure transducer (Utah Medical Products Inc., catalog number: 650-950)
13. M3000 tabletop isoflurane, non-rebreathing anesthesia machine (Supera Anesthesia Innovations)
14. Heated small animal operating table (Harvard Apparatus, catalog number: 50-1247)

## Software

1. Lab Chart Pro Version 7.3.8 (ADInstruments)

## Procedure

**Figure 18.**
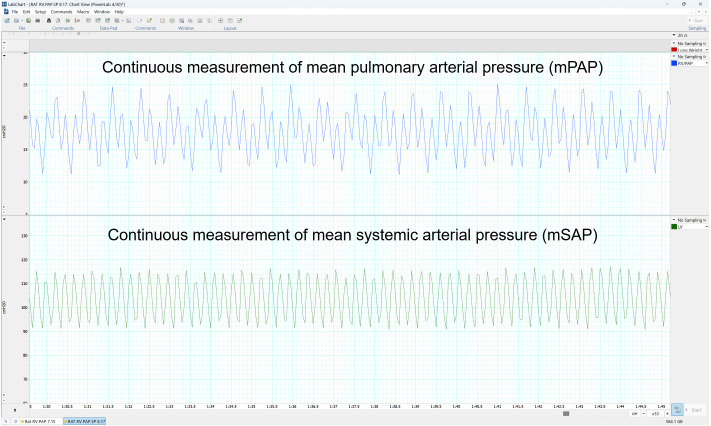
LabChart Pro Software showing the outputs as pressure curves

- **Preparation of PE-50 catheter for the carotid artery catheterization**
  1. Cut a 12-inch piece of PE-50 catheter with Vannas spring scissors (Figure 3A).
  2. Carefully insert a beveled 1 inch 25 G needle on the blunt end of the PE-50 catheter (Figure 3B).
*Note: If the catheter punctures during insertion, redo steps A1 and A2. The outer diameter of the 25 G needle is smaller than the inner diameter of the PE-50 catheter. When appropriately inserted, the needle and catheter can fit snugly without perforating the latter.*
  3. Prepare a slanted catheter end by cutting the other end of the catheter at a 45° angle (Figure 3C) with Vannas spring scissors.
*Note: The slanted end of the catheter is to be inserted into the right carotid artery.*
    **Figure 3.**
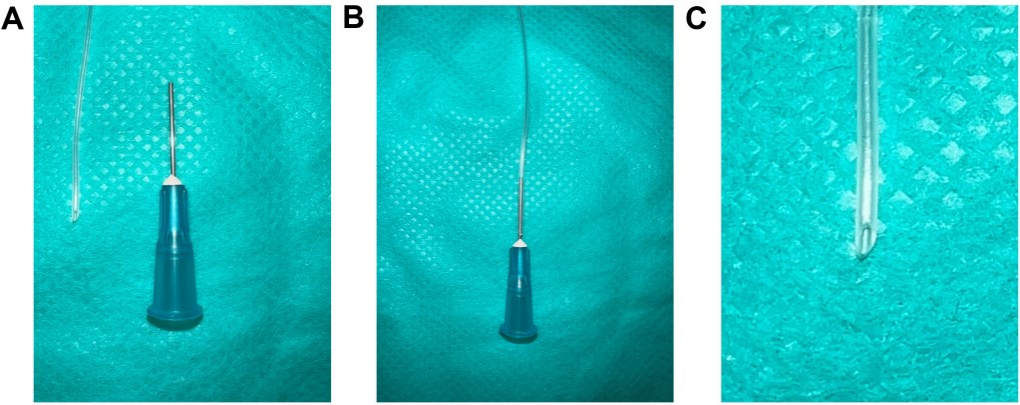
PE-50 catheter preparation for the carotid artery catheterization. (A) A 12-inch piece of PE-50 catheter with Vannas spring scissors and a 25 G needle, (B) Insert the 25 G needle into any end of the PE-50 catheter, (C) Zoomed-in view of the other end of the PE-50 catheter cut at 45° angle with Vannas spring scissors.
- **Preparation of umbilical venous catheter (UVC) for RHC**
  1. Bend 3.3 cm of 16 G beveled needle to a curve at a 65° angle (Figure 4A).
*Note: The main pulmonary artery arises from the right ventricle and curves posteriorly and slightly to the left before branching into the left and right pulmonary arteries. The 65° angle bending of the needle and the catheter mimics the curved pulmonary artery.*
  2. Insert 2.8 cm of the 3.5 French single lumen polyurethane UVC into the curved needle (Figure 4B).
*Note: Proper orientation of the curved needle during catheterization is of utmost importance, with the number markings on the UVC being required to face upwards during insertion of the needle. Furthermore, during insertion of the catheter into the right jugular vein, the orientation will ensure that the curvature of the catheter is parallel to the number markings, permitting the surgeon to determine the insertion length of the UVC.*
  3. Submerge the UVC containing the bent needle in 45 °C deionized water for 2 min.
  4. After 2 min, remove the UVC containing the bent needle from the 45 °C deionized water and place it in room temperature deionized water immediately.
  5. Remove the catheter from the needle; upon taking it out from cold water, the shape of the UVC should conform to the shape of the bent needle (Figure 4C).
*Note: To reuse the curved UVC, keep the curved needle inserted inside the curved catheter to maintain the curvature, as shown in Figure 4B. It is critical to ensure the curved catheter retains the 65° to avoid missing the pulmonary artery during insertion, which can lead to reinsertions and the eventual puncturing of the artery.*
    **Figure 4.**
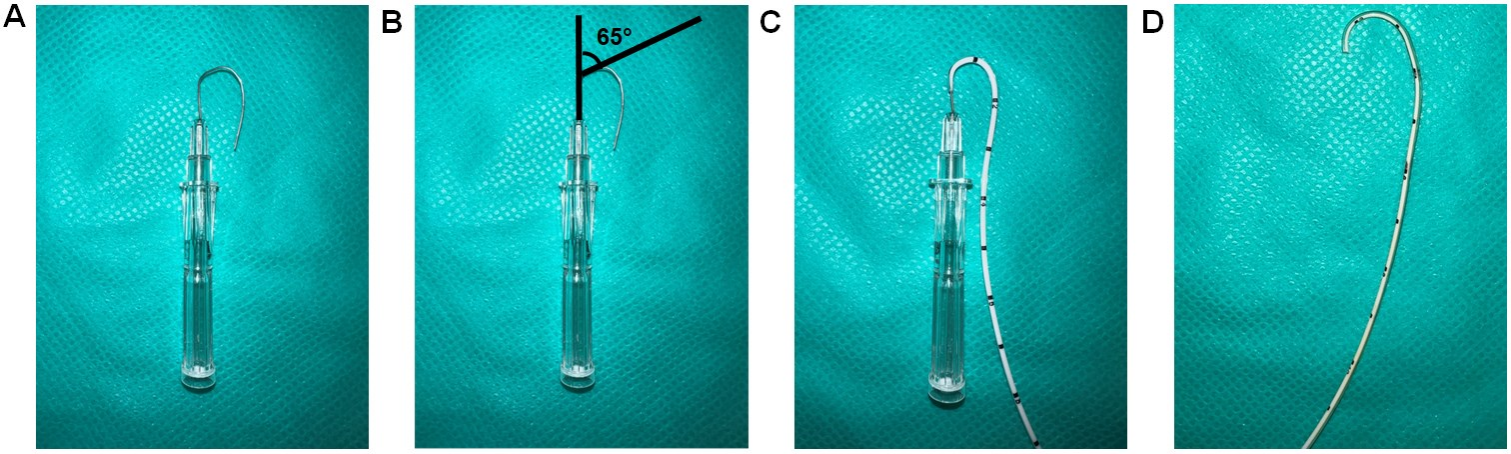
Preparation of a curved catheter. (A) Bend 3.3 cm of a 16 G needle to 65°. (B) Diagram showing the 65° bend. (C) Insert 2.8 cm of UVC into the needle. (D) The catheter is curved by placing a curved needle with the catheter in 45 °C deionized water for 2 min and then putting immediately into room-temperature deionized water.
- **Steel suture as guide wire setup for RHC (assemble specifically for step H6a)**
  1. Attach a 3-way stopcock to a pressure transducer (Figure 5A).
  2. Attach the 3.5 French dual lumen silicone UVC to the 3-way stopcock (Figure 5B).
  3. Insert the surgical stainless-steel suture via the 3.5 French dual lumen silicone UVC up to the end of the catheter tip and tighten the knob to secure the steel suture (Figure 5C).
    **Figure 5.**
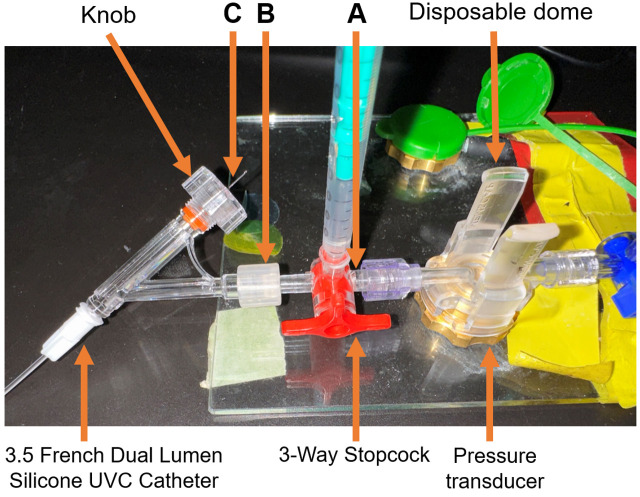
Steel suture for guide wire setup. (A) A 3-Way Stopcock attached to a pressure transducer. (B) 3.5 French Dual Lumen Silicone UVC attached to a 3-Way Stopcock. (C) Surgical stainless-steel suture inserted through the 3.5 French Dual Lumen Silicone UVC up to the end of the catheter tip and tightened knob, to firmly secure the steel suture. Insert the surgical steel into UVC through the knob and ensure the steel is fully inserted but not protruding out of the other end. The excess steel wire must hang from the knob.
- **Catheter sleeve preparation for RHC (assemble specifically for step H6b)**
  1. Open the Safelet IV catheter 20 gauge 1" Luer tapered end Teflon from its packaging (Figures 6A and 6B).
  2. Detach and remove the needle from the uncut catheter sleeve (Figure 6C).
*Note: The needle may be safely disposed of, as it does not serve any further purpose for catheterization. The needle protector, on the other hand, functions as a sleeve for the UVC*
  3. Cut the syringe fitting with scissors from the top and trim the tip of the catheter sleeve (Figure 6D).
  4. Insert the French single lumen polyurethane UVC into the catheter sleeve until the tip of the catheter is aligned with the tip of the catheter sleeve (Figure 6E–6G).
    Sections C and D are two different methods to perform the right heart catheterization. Irrespective of the method chosen, the data collected will be similar since all these methods lead the UVC to the pulmonary artery.
    **Figure 6.**
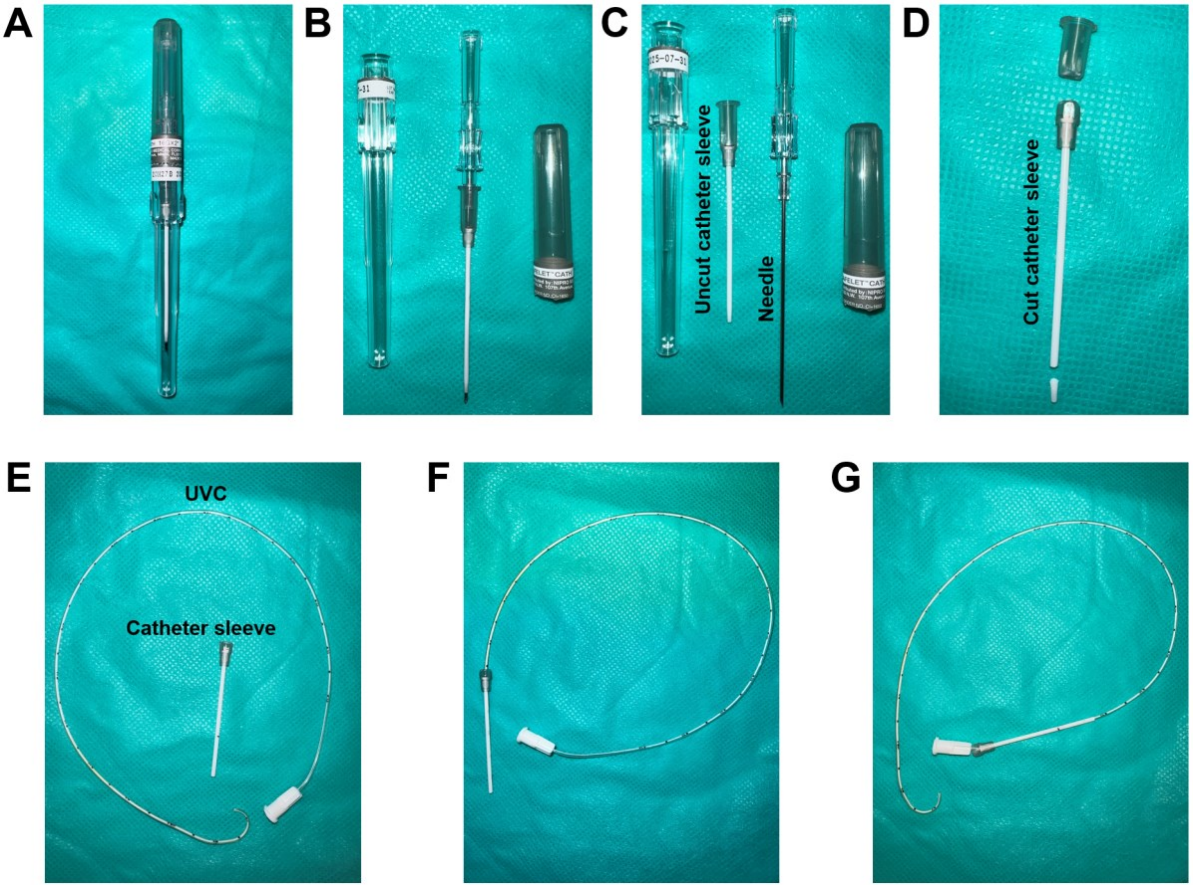
Catheter sleeve preparation. (A) Safelet IV Catheter 20 Gauge 1" Luer Tapered End Teflon in packaging. (B) Safelet IV Catheter 20 Gauge 1" Luer Tapered End Teflon open tube. (C) Detach the needle from the protecting sleeve. (D) Cut the syringe fitting from the top and trim the tip of the catheter sleeve. (E) The prepared catheter sleeve and the bent UVC. (F) Insert the UVC into the catheter sleeve until the tip of the catheter is aligned with the tip of the catheter sleeve. (G) Once the catheter sleeve is inserted into the right jugular vein, the catheter sleeve can be retracted leaving the bent part of the catheter inside the vein.
- **Setup of pressure transducer sensors and calibration with PowerLab Data Acquisition system**
  1. Setup of PowerLab system (Video 1)
    PowerLab system converts the signals received from the rat arteries into numerical data for analysis. One end of the catheter is inserted into the carotid or the pulmonary artery of the rat, and the other end of the catheter is connected to a dome attached to a pressure transducer sensor. The pressure changes in the carotid or pulmonary artery of the rat cause domes to deform, and the pressure transducer senses the deformation to determine the pressure. The pressure transducer converts the pressure into electrical signals and transmits the data to the bridge amplifiers. Bridge amplifiers connect the pressure transducer to the PowerLab, which delivers all data to the computer equipped with Lab Chart Pro Software that shows the outputs as pressure curves.
    **Video 1.**
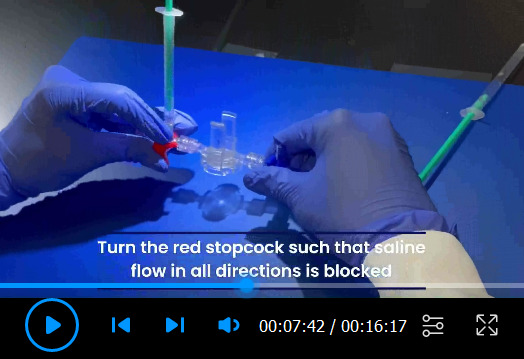
Setup of pressure transducer sensors and calibration with PowerLab Data Acquisition System
    - Connect the PowerLab system to a USB port of a computer and then connect the PowerLab system to the power outlet.
    - Connect two bridge amplifiers to the PowerLab. For each rat, use two bridge amplifiers to measure mPAP and mSAP—one for mPAP and another for mSAP—at the same time. For each bridge amplifier, connect the output port of the bridge amplifier to the input port of the PowerLab and connect the input port of the amplifier to the MLT1199 SP844 Kit (Figure 7A).
*Note: The PowerLab has 16 input ports; thus, each PowerLab can support up to 16 bridge amplifiers. Two amplifiers, one for measurement of mSAP and another for mPAP are required for each rat; therefore, a PowerLab can support up to eight rats for simultaneous measurement of mPAP and mSAP.*
    - Attach the bottom of the pressure transducer sensors to the acrylic sheet with a hot glue gun. For each rat, two pressure transducer sensors (one for mPAP and one for mSAP) are attached to the acrylic sheet (Figure 7B).
    - Label each bridge amplifier that is connected to input in transducer sensor and output connected with PowerLab wires with the PowerLab port number. For example, if the bridge amplifier is connected to PowerLab Port #1, label the bridge amplifiers, and its input and output wires as 1 (Figure 7C).
      **Figure 7.**
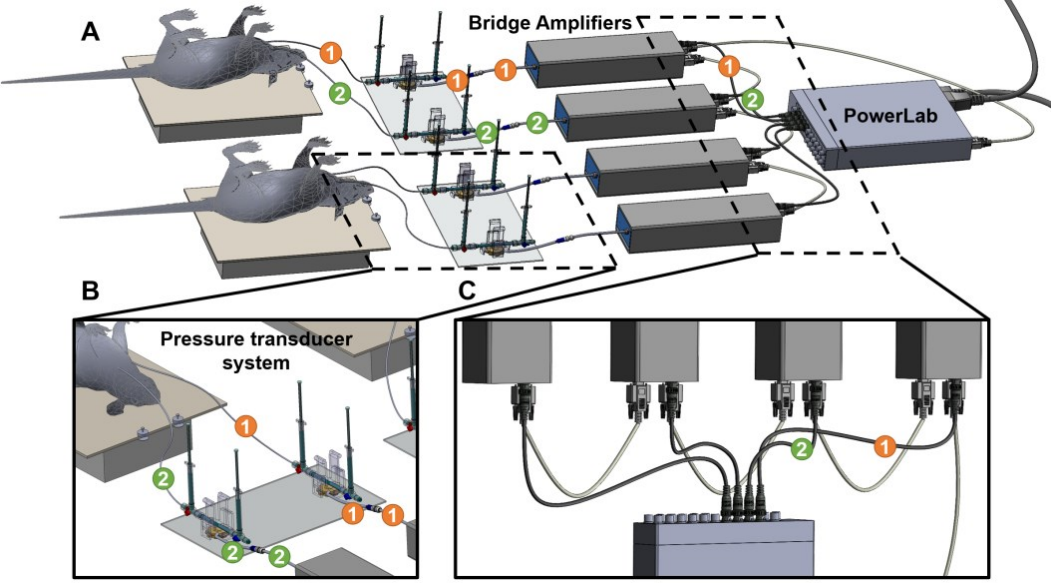
Setting up PowerLab, Bridge amplifiers, and transducers. (A) Overall setup of the equipment for simultaneous measurement of mean systemic arterial pressure (mSAP) and mean pulmonary arterial pressure (mPAP). (B) Zoomed-in view of two pressure transducers attached to a plexiglass plate and the domes with their respective 3-way stopcocks attached. One catheter is inserted in the carotid artery and the other in the jugular vein from the 3-way stopcocks. The transducers are connected to the bridge amplifier. (C) The first connection is between the PowerLab, and the bridge amplifier goes directly from the Power lab to the first bridge amplifier. Each consecutive bridge amplifier is also interconnected with digital input output cables (provided by AD Instruments with the PowerLab). The PowerLab is also the energy source for the bridge amplifiers; therefore, all the bridge amplifiers are connected to the front-end interface of the PowerLab system.
  2. Setup of manual fluid (saline) calibration (Video 1)
    - Attach one red 3-way stopcock with its female Luer lock inserted in the male swivel Luer lock of the disposable clip-on dome, and attach one blue 3-way stopcock with its male swivel Luer lock inserted on the female Luer lock side of the dome (Figure 8).
*Note: Male Luer swivel lock of the dome connects to the female Luer lock side of the 3-way stopcock.*
      **Figure 8.**
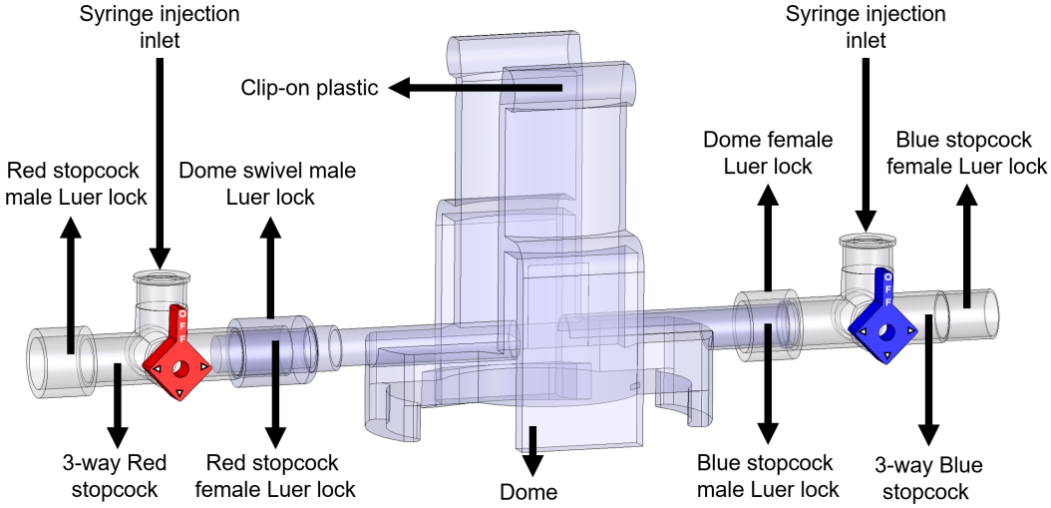
Components and connections of the disposable clip-on dome and 3-way stopcocks are used to connect catheters to the PowerLab system and catheters
    - Turn the red stopcock knob such that the knob blocks saline flow through the *left outlet* (Figure 9A), which forces the saline into the dome, towards the blue stopcock. Turn the blue stopcock knob to allow saline to flow in all directions except upwards (Figure 9A).
    - Fill a 1 mL HSW syringe with 0.9% (w/v) isotonic saline and place it in the inlet of the red stopcock (Figure 9A). The syringe does not require a Luer-lock connection. Press down so that saline flows into the dome (make sure no air bubbles are inside the transducer) until it comes through the *right outlet* of the blue stopcock. Then, turn the red stopcock knob to restrict saline flow in all directions (Figure 9B).
*Note: If the red stopcock knob is not restricted, removal of the syringe will induce air bubbles inside the transducer.*
    - Remove the syringe from the inlet of the red stopcock, refill it with saline, and place the syringe in the inlet of the blue stopcock (Figure 9C). Turn the blue knob to prevent flow towards the dome (Figure 9C) and press down the piston until saline comes out of the *right outlet* of the blue stopcock (make sure no air bubbles are inside the blue stopcock). Turn the blue stopcock knob (Figure 9D) to restrict saline flow in all directions and leave the syringe in the inlet of the blue stopcock.
    - Use a second syringe and fill it up with saline. Turn the red stopcock knob to allow saline to flow in all directions except towards the dome (Figure 9D) and place the syringe in the inlet of the red stopcock. Press the piston down until saline comes out through the *left outlet* (make sure no air bubbles are inside the red stopcock). Leave the syringe in the inlet of the red stopcock.
    - Insert the dome on a transducer sensor and turn the blue stopcock knob to allow saline to flow in all directions except upwards (Figure 9E). The pressure inside the dome is at 0 mmHg now, which is atmospheric pressure.
      **Figure 9.**
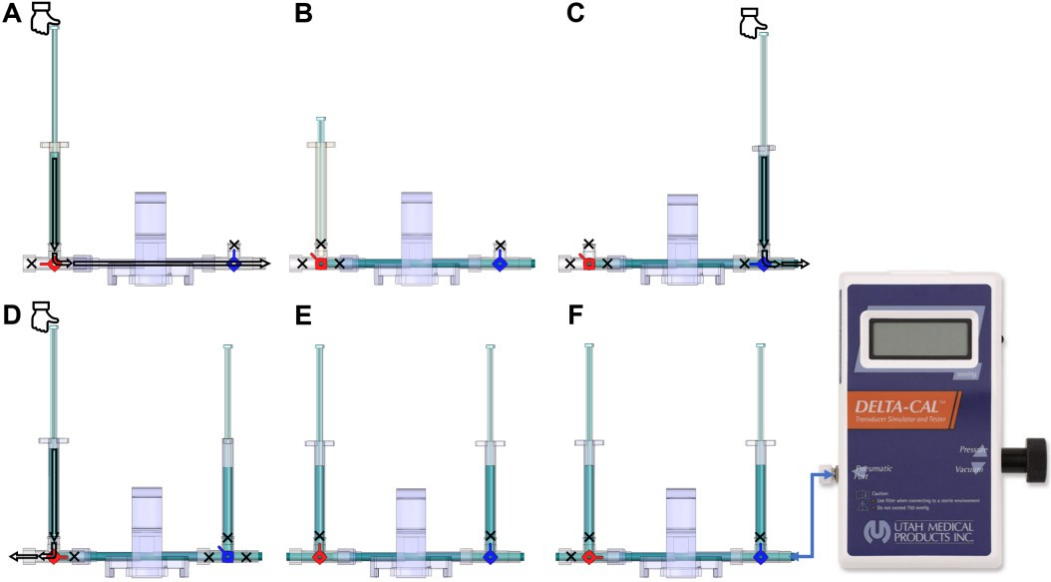
Dome saline calibration process. (A) This red knob orientation restricts the saline from flowing to the left of the red 3-way stopcock. This orientation allows the saline from the syringe to flow towards the blue valve. (B) After pressing the syringe, the saline flows towards the right side (arrow); once the dome and the stopcocks are filled with saline, shown as blue region, turn the red knob. This will block (shown by the cross-sign) all the inlets of the red 3-way stopcock and allow the liquid to stay within the dome without any leaks when the syringe is removed. (C) Place a saline-filled syringe on the blue 3-way stopcock, press down to fill all the regions of the blue stopcock with saline, and leave the syringe attached. (D) Place another saline-filled syringe on the red 3-way stopcock, turn the red knob in the orientation shown to restrict flow into the dome, and fill up the rest of the red 3-way stopcock to be entirely filled with saline. (E) After the previous step, the dome is ready to be placed on the sensor for 2-point calibration. Follow this orientation of the knob to do zero calibration explained in step E3b. (F) Use this orientation of knobs to conduct 2-point calibration and attach the Delta-Cal male swivel Luer lock to the female Luer lock of the blue 3-way stopcock, as explained in step E3c.
  3. Manual fluid (saline) two-point calibration of the PowerLab system using Lab Chart Pro (Video 1)
    - Turn on the PowerLab and open the Lab Chart Pro software.
    - Attach the Delta-Cal Pressure transducer male swivel connector to the blue stopcock female port (Figure 9F) with the stopcock orientation shown in Figure 9E. The stopcock orientation in Figure 9E is used for zero-calibration only. Turn on the Delta-Cal and the pressure reading should be 0. In Lab Chart Pro, click the Start button to start the continuous measurement of the sensor. Under the channel name where there is a plain white region, right-click and select Bridge Amp. Now click the Zero button to zero calibrate the sensor.
*Notes:*
*i. After turning on Delta-Cal, the reading may not be 0 if any of the stopcocks block the flow within the dome, which builds pressure in the dome.*
*ii. Make sure there are no vibrations or movements near the sensor during calibration.*
*iii. Zero function allows the sensor to identify when the electrical signal is at 0 V and the pressure is also at 0 mmHg.*
    - Now, turn the red knob of the red 3-way stopcock to prevent upwards and leftwards flow and retain the blue knob of the blue 3-way stopcock orientation (Figure 9F). This will allow the liquid to be confined within the dome when pressure is applied. The stopcock orientation in Figure 9F is to be used when calibrating at 50 mmHg and 120 mmHg, explained in later steps.
*Note: This setting keeps liquid inside the dome; when pressure is applied, the diaphragm in the middle of the dome will expand upwards but the volume of saline in the dome will remain the same.*
    - Go to Setup and then Channel settings. This window shows all 16 channels that will be collecting data (Figure 10). On the second column of the window, select by clicking the number of active channels. Channel 1 corresponds to the bridge amplifier connected to port 1 in PowerLab, and so on. Input the number of channels on the bottom right side of the window, based on the total number of active channels. Press OK to continue.
*Notes:*
*i. It is important to change the input for the number of channels based on active channels, or else the software will show all the channels.*
*ii. Do not change any other parameters on the window, such as the sample rate and range, until after calibration.*
      **Figure 10.**
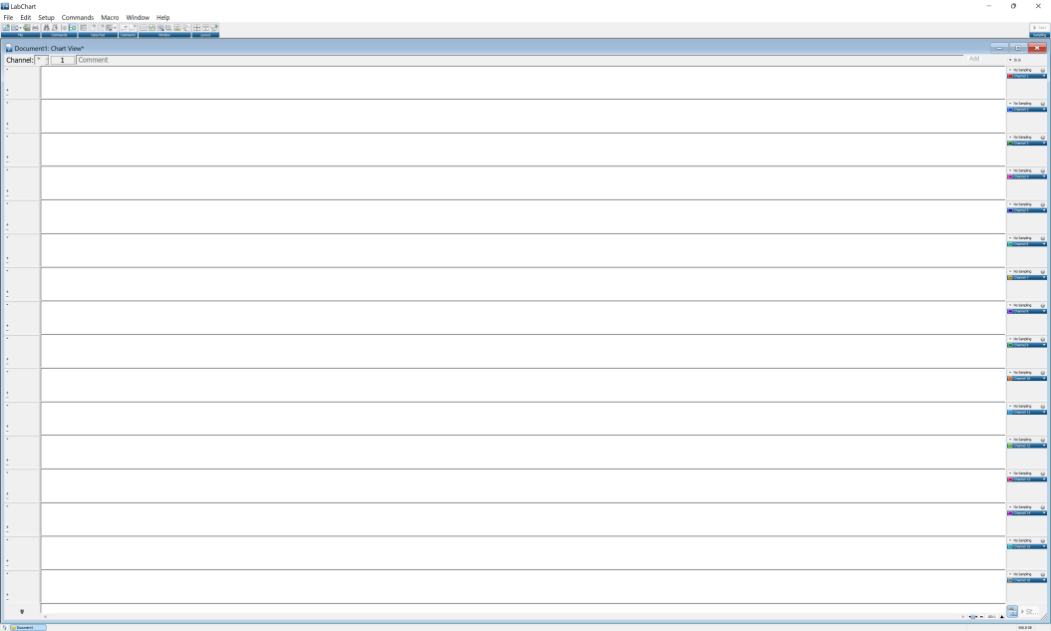
LabChart Pro window showing all 16 channels of the PowerLab Data Acquisition System
    - In the Lab Chart Pro, use the scale button on the bottom right of the screen and change it to 100:1 or 200:1.
*Note: Scaling allows x-axis values of the graph (time) to be within the window for two-point calibration. Using the computer mouse, select the region that covers both parts of the chart.*
    - On the left unselected region adjacent to the continuous measurement, right-click and select Auto Scale.
*Note: Auto Scale all the y-values of the graph (current with Amps unit) to be within the window for two-point calibration.*
    - Now, turn the Delta-Cal knob clockwise to increase the pressure to 50 mmHg. In the comment section of Lab Chart Pro, type 50 and press Add. This will mark the point in the data to indicate when the pressure was 50 mmHg. Allow the measurement of data at 50 mmHg for at least 3 s. Increase the pressure in the Delta-Cal to 120 mmHg by turning the knob clockwise, type 120 in the comment section, and press Add to indicate that data point corresponds to 120 mmHg pressure; allow at least 3 s of continuous data measurement at 120 mmHg.
    - Click Stop in the bottom right part of the screen to stop data collection. Select the region covering both parts in the graph (Figure 11A).
    - Under the channel name, you will see a plain white region; right-click and select Unit Conversion. On the left bottom side of the window, click the plus and minus signs until the selected region is in view.
*Note: The entire graph should be visible in the -6 mV to 6 mV range.*
    - Click on Units and select mmHg. Now, select the region corresponding to 50 mmHg (Figure 11B) and click the arrow next to Point 1. This will input an average of the selected region values. Then type 50 in the box adjacent to it (Figure 11C).
    - Now, select the region corresponding to 120 mmHg (Figure 11D) and click the arrow next to Point 2. This will input an average of the selected region values. Then, type 120 in the box adjacent to it (Figure 11E) and press OK. This completes the two-point calibration (Figure 11F).
    - To verify the calibration, click Start for continuous data collection. The number (mmHg) on the top of the channel name should be the same as the number (mmHg) on Delta-Cal. You may decrease the pressure in Delta-Cal to 80 mmHg and check if it shows 80 mmHg on the computer screen.
*Note: An error of ± 1 is acceptable due to machine error.*
    - Repeat steps E3c–E3m for each pressure transducer sensors, since one transducer requires one channel feedback in the Lab Chart Pro.
      **Figure 11.**
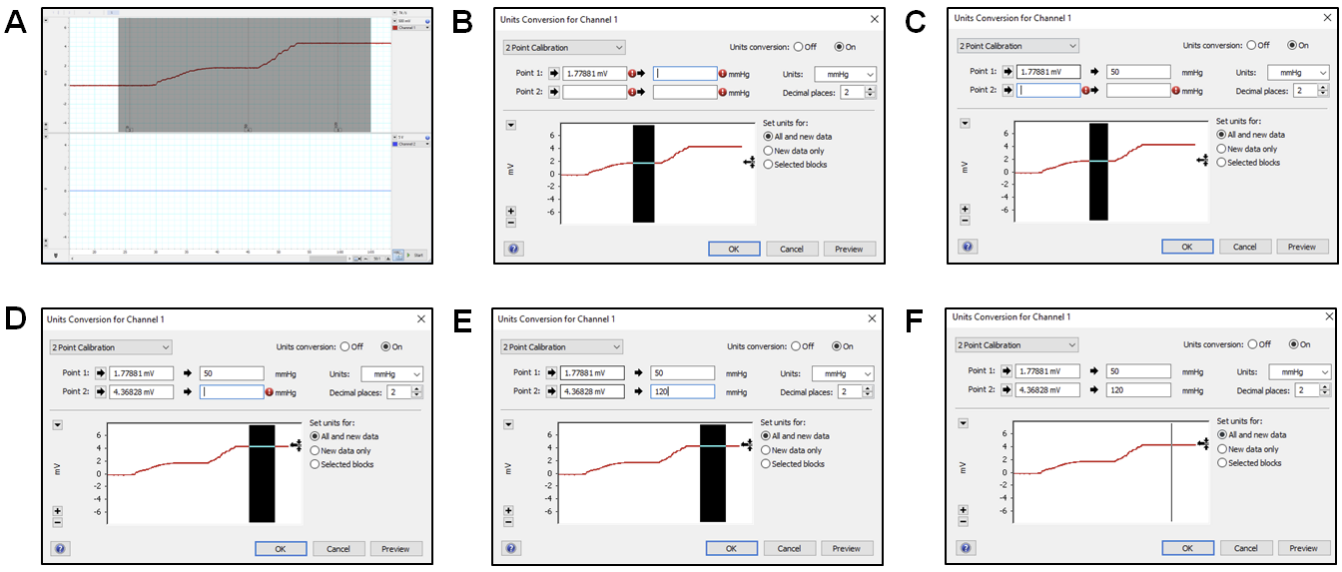
2-point calibration using Lab Chart Pro. (A) Selecting the region from the collected continuous measurement for unit conversions using the 2-point calibration method. (B) Select the region that shows continuous measurement at 50 mmHg and click on the arrow next to *Point 1* to average the values selected and type that value. (C) Type *50* next to the second arrow for *Point 1* to allow the sensor to convert the value in the first box to reflect 50 mmHg. Change the *Units* box to *mmHg.* (D) Select the region that shows continuous measurement at 120 mmHg and click on the arrow next to *Point 2* to average the values selected and input that value. (E) Type *120* next to the second arrow for *Point 2* to allow the sensor to convert the value in the first box to reflect 120 mmHg. (F) Unit conversion window for completed 2-Point calibration.
    - A PE-50 catheter or UVC heads can now be fit into the red stopcock male port, with the red knob allowing saline flow to the left and right but no flow upwards and the blue knob turned such that there is no flow (Figure 12A). Press the syringe filled with saline to allow the catheters to be pre-filled with saline. To begin measurement of mPAP and mSAP, match the knob orientation shown in Figure 12B, insert the other end of the catheter into the jugular vein or the carotid artery.
      **Figure 12.**
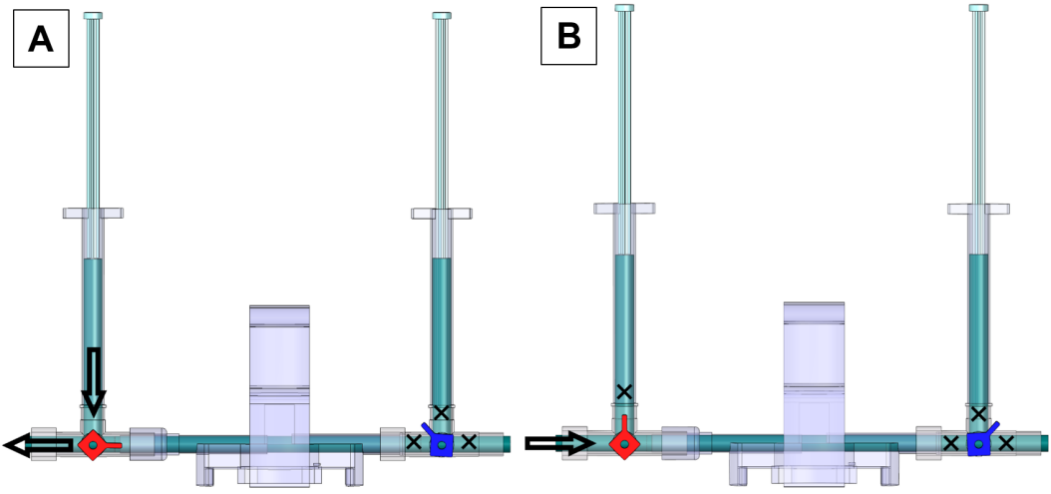
Valve orientations to pre-fill saline and begin measurements of mPAP and mSAP. (A) Valve orientation for catheter ready to pre-fill with saline: turn the red knob such that flow from the left syringe to the left outlet of the red stopcock is allowed and turn the blue knob such that flow is restricted in all directions. (B) Valve orientation for catheter ready to begin measurement. Turn the red knob such that the upwards flow is blocked and turn the blue knob such that flow in all directions is blocked. The arrow indicates the female red knob port that attaches to a PE-50 catheter or UVC.
- **Preoperative procedures**
  1. Prepare surgery tray with the tools shown in Figure 2 before surgery. Clean all tools with soap and water and wipe them with 70% isopropyl alcohol.
  2. Place the heating pad on a flat surface under the inverted light microscope and clean the heating pad surface with 70% isopropyl alcohol.
*Note: The light microscope helps to better visualize the arteries and catheterization.*
  3. Turn on the heating pad and set the temperature to 37 °C.
*Note: Maintain the temperature at 37 °C throughout the surgery and experiment.*
  4. Place the rats in the inhalation anesthesia chamber with 2.5% isoflurane. Once the rat falls asleep, inject intraperitoneally 300 μL per 250 g rat body weight with a cocktail of 1 mL of 100 mg/mL ketamine and 100 μL of 20 mg/mL xylazine. Confirm anesthesia by the toe-pinch method. More anesthesia can be administered in small increments if sufficient induction is not obtained with the initial dose.
*Note: One dose of intraperitoneal anesthesia will keep the rat anesthetized for at least one hour. To ascertain the state of unconsciousness, the toe-pinch method may be utilized at any point of the surgery. Sudden movements in the rat should be avoided. However, any muscle movements or agitation observed in the rat during the surgical procedures are an indication that the rat is beginning to regain consciousness. In such cases, an additional dose of anesthesia should be administered.*
  5. Place the rat in the supine position on the heating pad (Figure 13A). Restrain all four legs with micropore tape and the head by placing a string under the upper incisors, as shown in Figure 13A.
  6. (Optional) Using a hair trimmer, remove hair from the ventral neck and the dorsal area between the scapulae.
  7. Scrub the trimmed area with Betadine^®^ and then with 70% isopropyl alcohol for three cycles.
*Note: Steps F6 and F7 are optional for experienced researchers but recommended for those who are new to the process.*
    **Figure 13.**
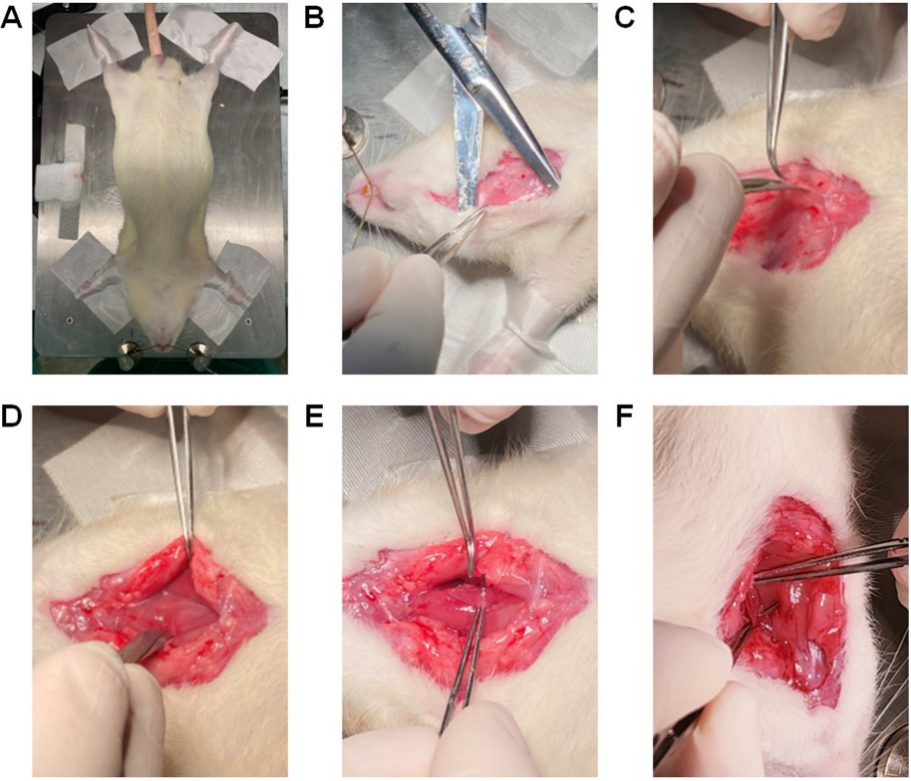
Restraining the rat and exposing the ventral neck area for catheterization of the left carotid artery. (A) Position the rat with the chest facing upwards and the head towards the surgeon on the heating pad. (B) Using straight Mayo scissors, make an incision of ~3 cm at the middle line of the ventral neck area and use the scissors as pliers to detach the skin from the subcutaneous tissues. (C) Adjacent to the left side of the trachea, go deeper by cutting the connective tissues and muscles with tenotomy scissors for coarse cutting. (D) Separate the muscle layers to cut deeper into the left side of the trachea until the nerve and the carotid artery is exposed. (E) Using curved tip Graefe forceps, gently separate the nerve that is attached to the left carotid artery. (F) The carotid artery thus exposed and free from innervation is used for catheterization.
- **Catheterization of the left carotid artery (Video 2)**
  **Video 2.**
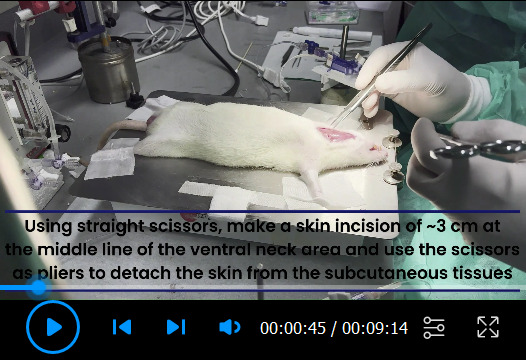
Left carotid artery for mean systemic arterial pressure (mSAP). The animal studies were performed according to the guidelines from the University of Illinois at Chicago approved by the Animal Research Ethics Board of University of Chicago under the protocol #: ACC21-180.
  1. Using straight Mayo scissors, make a skin incision of ~3 cm at the middle line of the ventral neck area and use the scissors as pliers to detach the skin from the subcutaneous tissues (Figure 13B). Adjacent to the left side of the trachea, go deeper by cutting the connective tissues and muscles with tenotomy scissors for coarse cutting and spring scissors for finer cutting, until the left carotid artery is visible (Figure 13B–13D).
*Notes:*
    - *Be careful not to puncture or cut the trachea and the left carotid artery while removing tissues and muscles.*
    - *Minor bleeding may appear due to cutting small blood vessels in the area. So, apply a gauze pad to control the bleeding or cauterize vessels using the cautery kit.*
  2. Using Graefe forceps, gently separate the nerve around the left carotid artery (Figure 13E).
*Notes:*
    - *Use the microscope to locate the left carotid artery to avoid severing surrounding blood vessels or nerves.*
    - *Make sure that the nerve is separated from the carotid artery (Figure 13F).*
  3. Place a Graefe forceps under the left carotid artery (Figure 14A), lift it up, and keep it exposed for surgical manipulation. Leave the Graefe forceps towards the left side of the rat body away from the trachea.
*Note: If the forceps is on the top of the trachea, it may choke the rat.*
  4. Using a 5-0 suture, tie one end of the carotid artery (towards the head) by making a double knot and then a single knot (Figure 14B) to stop the blood flow to the brain. Place a micro serrefine on the other end of the carotid artery (towards the heart) (Figure 14C).
*Notes:*
    - *Use the microscope to assess whether the artery is throbbing; if it is, placing a micro serrefine should help reduce artery throbbing.*
    - *Usually, lifting the artery with Graefe forceps (in step G2) prevents blood flow and throbbing.*
    - *Do not cut the excess suture, because excess suture will be used to anchor the catheter to the carotid artery in step G8.*
  5. Using a 5-0 suture, leave an open double knot on the other end of the carotid artery (towards the heart) but before the micro serrefine (Figure 14D).
*Note: The open double knot must not be tied before inserting the catheter through the left carotid artery but tied after insertion of the catheter.*
  6. Using a micro dissecting spring scissor, make a ~0.5 mm hole in the carotid artery (Figure 14E).
*Notes:*
    - *~0.5 mm is slightly larger than the diameter of the catheter tip.*
    - *Be careful not to sever the carotid artery when making the hole using micro dissecting spring scissor.*
  7. Hold the PE-50 catheter (prepared in Section A) with angled vessel cannulation forceps (Figure 14F and Figure 14G), insert 1.0 cm of the catheter into the hole of the carotid artery (Figure 14H), and secure it with a piece of micropore tape (Figure 14I). Further secure the catheter in place by closing the open double knot, followed by tying a single knot on top of the inserted catheter (Figure 14J). Anchor the catheter to the carotid artery with the first suture (as stated in step G4) by making another double knot and then a single knot. Then, remove the micro serrefine (Figure 14K) and confirm the characteristic carotid artery peaks in the computer monitor (Figure 18). Now the mSAP can be measured.
*Note: The flushing of a catheter with saline solution is a crucial procedure to be carried out during arterial catheterization. It serves the purpose of clearing any blockages that may occur in the catheter channel due to the coagulation of blood. A pre-filled syringe containing saline solution is used to exert pressure on the catheter and remove any obstructions. The presence of a clogged catheter can lead to erroneous or non-existent feedback reaching the sensors, thereby compromising the accuracy of any measurements obtained from the catheter.*
  8. In addition, secure the dangling part of the catheter from the rat’s body to the transducer with a piece of micropore tape to the heating pad. These will further secure the catheter from unwanted movement.
  9. Place gauze pads to cover the exposed left side of the trachea and secure them with micropore tapes (Figure 14L).
*Note: Because this protocol entails continuous measurement of mSAP for at least 6 h, the exposed skin should be covered with gauze pads to prevent the area from drying.*
  10. Upon completion of all mSAP measurements, the PE-50 catheter should be flushed with a syringe containing 99% isopropyl alcohol. The catheter may be reused if it remains unpunctured and displays transparency after the cleaning procedure.
    **Figure 14.**
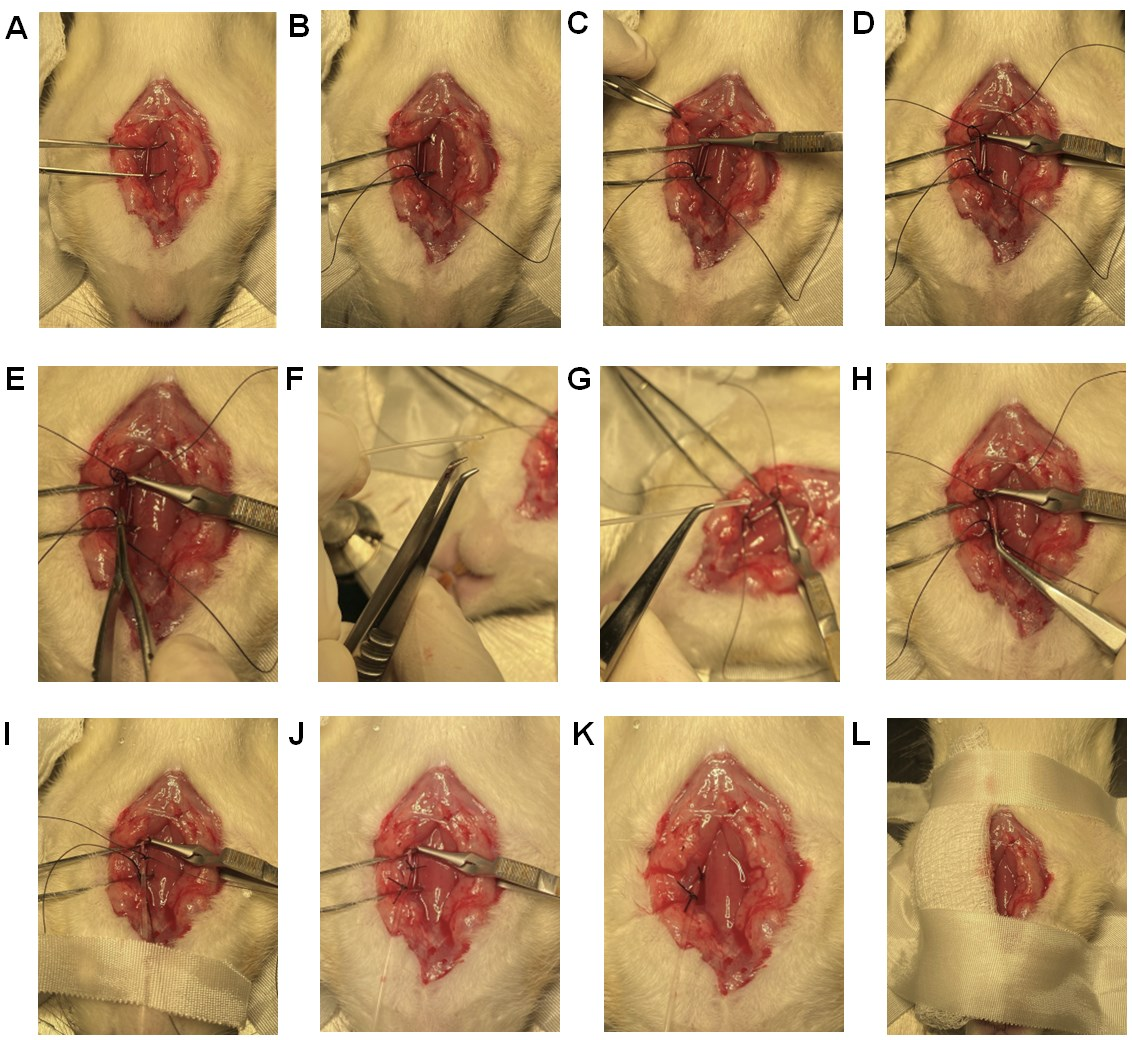
Catheterization of the left carotid artery. (A) Place one Graefe forceps under the left carotid artery to lift it up and keep it exposed for surgical manipulation. (B) Using a 5-0 suture, tie one end of the carotid artery (towards the head) by making a double knot and then a single knot. (C) Using the microscope, check whether the artery starts throbbing. If the artery is throbbing, place a micro serrefine on the other end of the carotid artery (towards the heart) to stop throbbing. (D) Using a 5-0 suture, leave an open double knot on the other end of the carotid artery (towards the heart) but before the micro serrefine. (E) Using a micro dissecting spring scissor, make a ~0.5 mm hole in the carotid artery. (F) This angled vessel cannulation forceps has teeth to grip the catheter. (G) Grab the prepared PE-50 catheter for the carotid artery with angled vessel cannulation forceps. (H) Insert 1 cm of the catheter via the hole in the carotid artery. (I) Secure the inserted catheter with a piece of micropore tape. (J) Secure the catheter in place by closing the open double knot followed by tying a single knot on top of the inserted catheter. Shorten all the sutures and clean any blood. (K) Remove the micro serrefine to allow the blood to flow and confirm the characteristic carotid artery peaks in the computer monitor. (L) Place gauze pads to cover the exposed left side of the trachea and secure them with pieces of micropore tape.
- **Catheterization of right jugular vein and pulmonary artery (Video 3)**
  Since this protocol involves simultaneous measurement of mSAP and mPAP, the right jugular vein of the same rat used to measure mSAP in Section G should be catheterized for measurement of mPAP.
  **Video 3.**
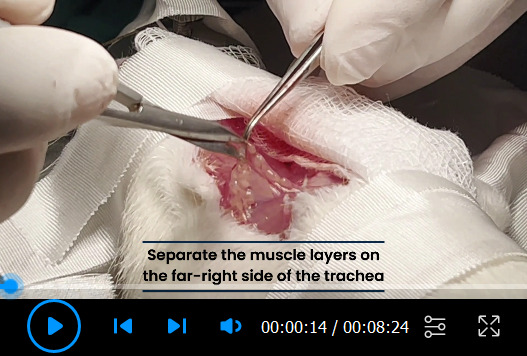
Right jugular vein for mean pulmonary arterial pressure (mPAP). The animal studies were performed according to the guidelines from the University of Illinois at Chicago approved by the Animal Research Ethics Board of University of Chicago under the protocol #: ACC21-180.
  1. Using tenotomy scissors for coarse cutting and spring scissors for finer cutting, separate the muscle layers on the far-right side of the trachea near the scapula, to find and expose the bifurcation of the right jugular vein (Figure 15A).
*Note: The same skin incision that was performed to expose the left carotid artery is used to locate and expose the right jugular vein.*
  2. Place a Graefe forceps under the vein below the bifurcation (Figure 15B) to lift the jugular vein up and leave the forceps to keep the vein exposed for surgical manipulation. As the vein goes towards the heart, the vein diameter gets larger, which is the area of interest.
*Note: You can see the bifurcation of the jugular vein at this step. Identification of the bifurcation is important for catheterization of jugular vein (Figure 15C).*
  3. Using a 5-0 suture, tie one end of the jugular vein (towards the head but below the bifurcation) by making a double knot and then a single knot that stops blood flow from the head area (Figure 15D).
  4. Using a 5-0 suture, leave an open double knot on the other end of the jugular vein (towards the heart) (Figure 15E).
  5. Grab the jugular vein, where the lumen diameter is larger, with another Graefe forceps and make a small incision of ~0.5 mm on the jugular vein with a micro dissecting spring scissor like the one shown in Figure 15E.
  6. Catheterization of the jugular vein can be performed using three different methods: (a) catheterization using steel guide wire can be used for rats with health issues or young rats and healthy adult rats; (b) catheterization using a catheter sleeve is recommended only for healthy adult rats; (c) catheterization using only the catheter is recommended for skilled surgeons.
    **Figure 15.**
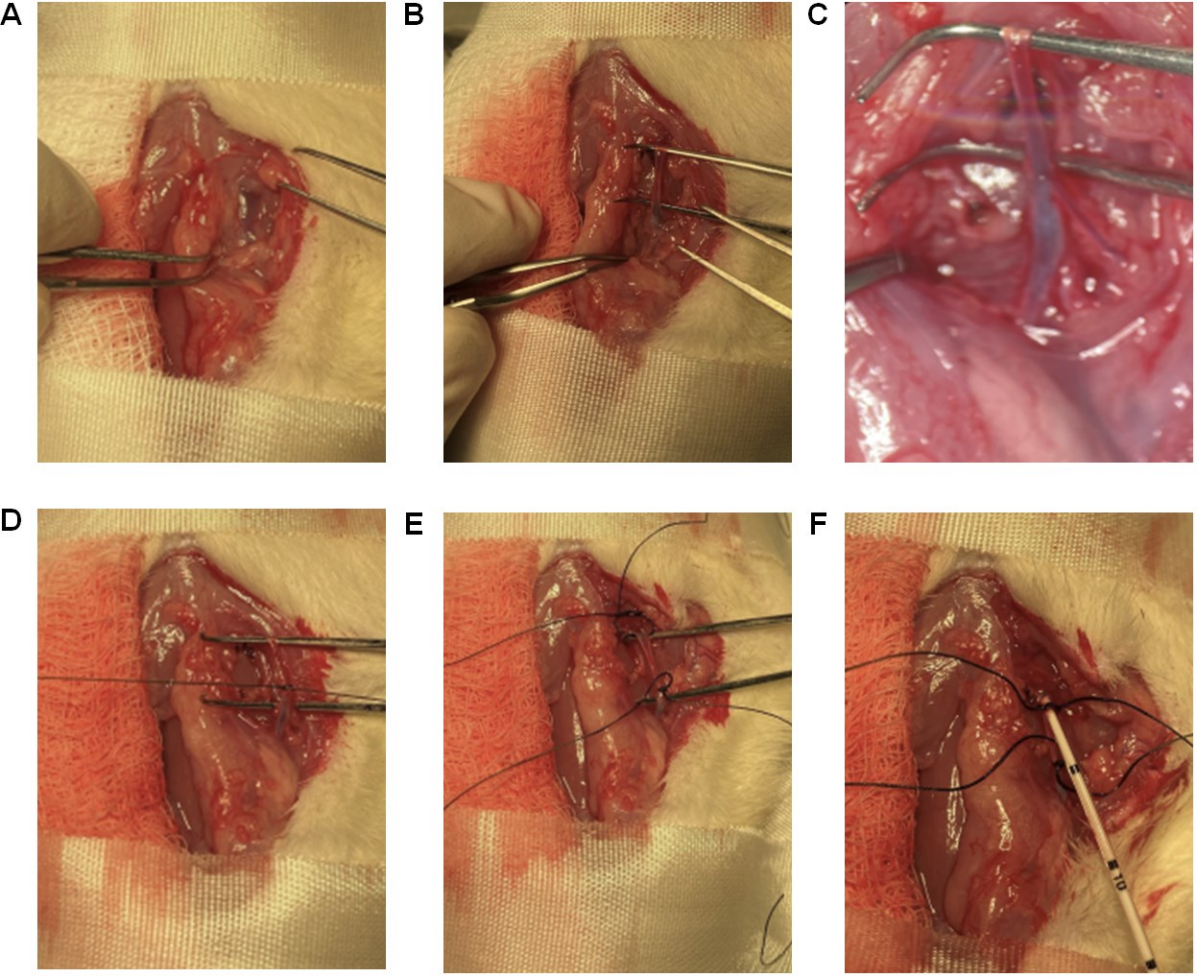
Catheterization of the right jugular vein. (A) Using tenotomy scissors for coarse cutting and spring scissors for finer cutting, separate the muscle layers on the far-right side of the trachea (near scapula) to identify and expose the bifurcation of the right jugular vein. (B) Place a Graefe forceps past the bifurcation (Fig. 12B) to lift the jugular vein up and keep it exposed for surgical manipulation. (C) Microscopic view of the bifurcation. (D) Using a 5-0 suture, tie one end of the jugular vein (towards the head but below the bifurcation) by making a double knot and then a single knot that stops blood flow from the brain. (E) Using a 5-0 suture, leave an open double knot on the other end of the jugular vein (towards the heart). (F) Slowly insert the catheter into the jugular vein for approximately 6–8 cm from the point of insertion, depending on the age of the rat.
    - Catheterization using steel suture as guide wire (corresponding to section C):
      i. Adjust the transducer to adapt to the steel suture as guide wire setup as discussed in section C.
      ii. Use the UVC with steel suture as guide wire using angled vessel cannulation forceps similar to that shown in Figure 14G.
      iii. With another Graefe forceps, hold one side of the hole made in the jugular vein for easy catheterization.
      iv. Slowly insert the catheter into the jugular vein for approximately 6–8 cm from the point of insertion, depending on the weight of the rat, in the specific orientation as shown in Figure 15F. If the weight of the rat is less than 250 g, insert the catheter 6 cm; if the rat weighs more than 250 g, insert 8 cm.
      v. Pull the steel suture outward to allow the curved catheter to conform to its original curved shape inside the vein.
    - Catheterization using a catheter sleeve (corresponding to section D) (method used in Video 3):
      i. Grab the catheter sleeve (Figure 16A) with the catheter using angled vessel cannulation forceps.
      ii. While keeping the jugular vein grabbed, insert 1 cm of the catheter sleeve into the jugular vein in the specific orientation as shown in Figure 16B.
      iii. Now insert the catheter in for approximately 6–8 cm from the point of insertion depending on the age of the rat.
      iv. While holding the catheter stationary (Figure 16C), pull the catheter sleeve out to the other end of the catheter.
      **Figure 16.**
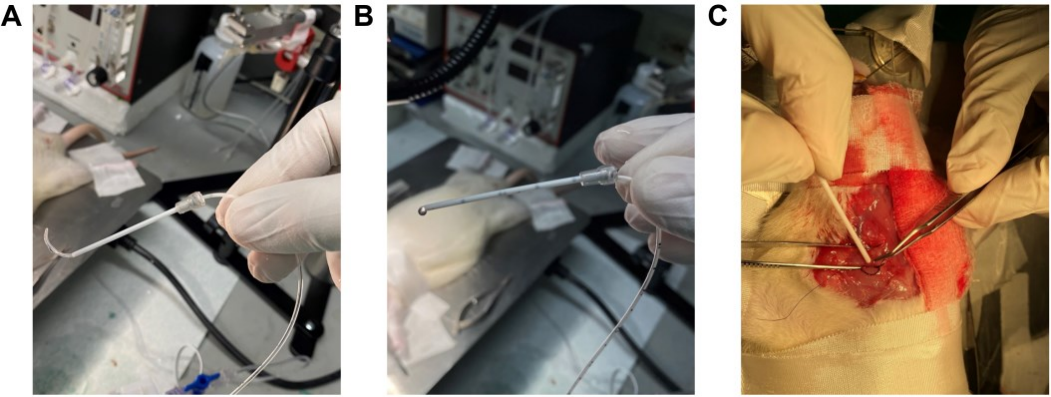
Using catheter sleeves for RHC. (A) Grab the bent UVC through the catheter sleeve. (B) Ensure that the catheter sleeve and the bent UVC are aligned with each other’s edges in this specific orientation with the black marks facing the surgeon. (C) Insert the catheter sleeve into the right jugular vein, and when 1 cm of the catheter is inside the vein, pull the sleeve backward leaving only the UVC inside the vein.
    - Catheterization using the UVC only:
      i. Grab the catheter using hollowed angled vessel cannulation forceps.
      ii. With extra fine Graefe forceps, grab one side of the hole made in the jugular vein for easy insertion of the catheter.
      iii. Carefully insert the catheter into the jugular vein for approximately 6–8 cm from the hole made, depending on the age of the rat, in the specific orientation as shown in Figure 15F.
*Note: When the curved catheter is inserted into the jugular vein, the shape of the catheter conforms to the vein; as it is maneuvered down the vein, the tip of the catheter starts to assume the curved shape, and once it reaches the pulmonary artery it assumes the original curved shape (60°–65° angle).*
  7. The pressure readings and characteristic peaks, displayed by the power system, change as the catheter is maneuvered through the right atrium, right ventricle, and then in the pulmonary artery. The pressure in the right atrium, right ventricle, and pulmonary artery should be 2–6 mmHg, 0–25 mmHg, and 10–25 mmHg, respectively, with their characteristic peaks (Figure 17).
  8. Once the characteristic peak of the pulmonary artery is confirmed, secure the catheter with a piece of micropore tape. Further, secure the catheter by closing the open double knot followed by tying a single knot on top of the inserted catheter. Now the mPAP can be measured.
  9. Place gauze pads to cover the exposed right side of the rat and secure them with micropore tapes.
*Note: Because this protocol entails the continuous measurement of mPAP for at least 6 h, the exposed skin needs to be covered with gauze pads to prevent dryness or adverse reaction due to long-time exposure to the environment.*
  10. After the completion of data collection, the rat should be euthanized using isopropyl alcohol. A syringe should be removed from the domes and filled with isopropyl alcohol. The alcohol-filled syringe should then be injected into the transducer, which will subsequently travel into the rat. A flat line measurement of mPAP or mSAP confirms rat euthanasia.
  11. Upon completion of all mPAP measurements, the UVC should be flushed with a syringe containing 99% isopropyl alcohol. The catheter may be reused as long as it remains unpunctured and displays transparency after the cleaning procedure.
    **Figure 17.**
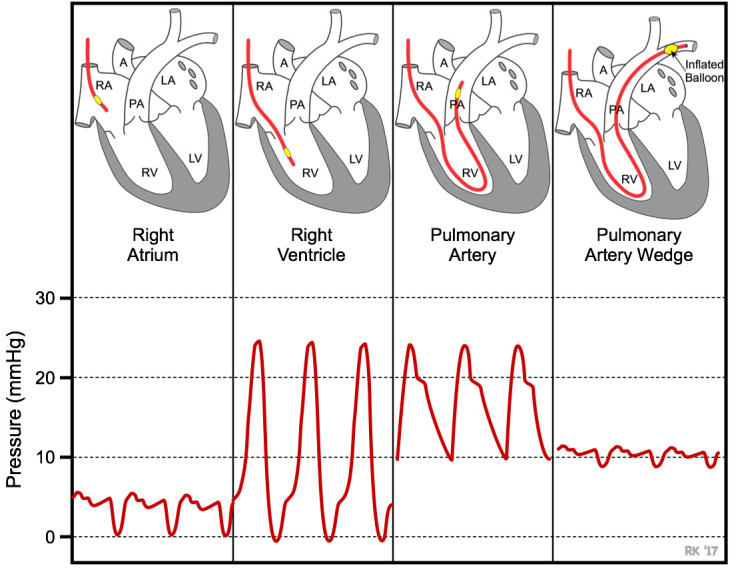
Characteristic peaks during RHC in the right atrium and right ventricle help to identify the location of the UVC as it maneuvered to the pulmonary artery. Used with permission from Dr. Richard Klabunde https://www.cvphysiology.com/Heart Failure/HF008.

## Data analysis

Lab Chart Pro collects mPAP and mSAP data in a continuous fashion every second (Figure 18). Data are first transferred to Excel for further processing.

1. Identify mPAP and mSAP data at baseline point or at zero time, as shown in Table 1 and Table 2.
  **Table 1.** Mean pulmonary arterial pressure (mPAP) data points of three groups: saline, pulmonary group; plain PGE, IV (120 μg/kg) group; and plain PGE, pulmonary (120 μg/kg), plotted in Figure 19A

Time (min)	Saline, pulmonary		Plain PGE, IV (120 μg/kg)		Plain PGE, pulmonary (120 μg/kg)	
	mPAP	% decrease	mPAP	% decrease	mPAP	% decrease
0	52.00	0.00	49.50	0.00	51.50	0.00
5	45.24	13.00	41.58	16.00	44.81	13.00
10	45.50	12.50	33.17	33.00	36.57	29.00
15	45.76	12.00	34.65	30.00	35.79	30.50
20	52.00	0.00	35.64	28.00	36.82	28.50
25	-	-	40.84	17.50	36.57	29.00
30	52.00	0.00	42.08	15.00	38.11	26.00
35	-	-	37.62	24.00	36.05	30.00
40	-	-	45.05	9.00	39.91	22.50
45	-	-	48.02	3.00	40.43	21.50
50	-	-	49.50	0.00	49.44	4.00
  **Table 2.** Mean systemic arterial pressure (mSAP) data points of three groups: saline, pulmonary group; plain prostaglandin E (PGE), IV (120 μg/kg) group; and plain PGE, pulmonary (120 μg/kg), plotted in Figure 19B

Time (min)	Saline, pulmonary		Plain PGE, IV (120 μg/kg)		Plain PGE, pulmonary (120 μg/kg)	
	mSAP	% decrease	mSAP	% decrease	mSAP	% decrease
0	115.00	0.00	119.00	0.00	118.00	0.00
5	112.13	2.50	73.19	38.50	89.09	24.50
10	108.68	5.50	63.07	47.00	75.52	36.00
15	109.25	5.00	64.26	46.00	75.52	36.00
20	109.83	4.50	69.02	42.00	79.06	33.00
25	110.98	3.50	80.92	32.00	91.45	22.50
30	109.25	5.00	80.33	32.50	94.40	20.00
35	112.13	2.50	95.20	20.00	97.35	17.50
40	117.30	-2.00	116.62	2.00	109.74	7.00
45	-	-	121.38	-2.00	109.15	7.50
50	-	-	121.38	-2.00	115.05	2.50
2. Spot and collect mPAP and mSAP at various time intervals (0, 15, 30, 60, 90, 120 to 240 min).
3. Plot mPAP and mSAP value against time.
4. Data can also be plotted in percentage decrease mPAP and mSAP, as shown in Figure 19A and Figure 19B. To calculate percent decrease, use the formula $\frac{Initial\;mPAP\left(0\;mins\right)-mPAP(a\;given\;time\;point)}{Initial\;mPAP(0\;mins)}\times 100\%$, based on initial mPAP, and the formula $\frac{Initial\;mSAP\left(0\;mins\right)-mSAP(at\;any\;time\;point)}{Initial\;mSAP(0\;mins)}\times 100\%$, based on initial mSAP. The mPAP (at a given time point) and mSAP (at a given time point) refer to any mPAP or mSAP values from the table at any time point, to find the percentage decrease at that time with respect to the original mPAP or mSAP, respectively.
  **Figure 19.**
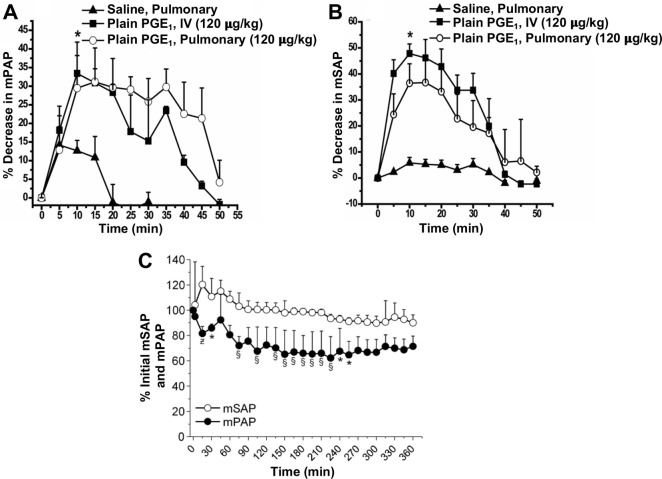
Acute pulmonary hemodynamic efficacy of plain prostaglandin E1 (PGE1) administered either intravenously (IV) or intratracheally (pulmonary). (A) mean pulmonary arterial pressure (mPAP) and (B) mean systemic arterial pressure (mSAP) after administration of plain PGE1. Data represent mean ± SD, n = 4–6; *p < 0.05.
